# Research on optimization of C4 repair operation of Harmony electric locomotive based on preventive maintenance

**DOI:** 10.1371/journal.pone.0328399

**Published:** 2025-07-24

**Authors:** Yihan Liu, Jieping Wu, Qiaoyu Wu

**Affiliations:** 1 School of Tianyou, East China Jiaotong University, Nanchang, China; 2 School of Transportation Engineering, East China Jiaotong University, Nanchang, China; Istinye University: Istinye Universitesi, TÜRKIYE

## Abstract

Harmonious High-Power AC Electric Locomotive, as the main type of locomotive for freight transport in China, the safety of its operation and maintenance issues have been the focus of great attention by the operation department. How to reduce the high maintenance cost and shorten the time-consuming long maintenance is a pressing issue for the operation management. In this paper, for the C4 repair operation of the harmonious electric locomotive, using the preventive maintenance method, we constructed a multi-component maintenance plan optimization model with the aim of maximizing the availability, and solved it by using a heuristic genetic algorithm. On this basis, component-specific maintenance schedules were developed based on reliability thresholds. The results of the study showed that the preventive maintenance approach resulted in a 51.04% reduction in total cost and a 7.89% increase in availability over the same operating cycle compared to the traditional fixed repair schedule maintenance program. Under the premise of ensuring locomotive operational safety, it effectively saves costs, reduces maintenance downtime and achieves the objective of improving the quality of C4 repairs.

## Introduction

With the rapid development of the railroad transportation industry, it has led to the continuous expansion of the railroad network and the increase of locomotive ownership. In 2024 China’s total rail freight traffic reached 5.175 billion tons. Locomotives in the process of high-intensity, long-distance operation, the frequency of use and operating mileage increased significantly, parts wear and aging speed up, which in turn led to maintenance and replacement needs continue to climb, making the locomotive maintenance-related workload increased significantly year by year. Traditional overhaul methods suffer from high costs, fixed maintenance intervals and over maintenance, making it difficult to find a balance between efficiency and quality. To meet this challenge, the locomotive maintenance industry needs to actively introduce advanced technologies and equipment and optimise maintenance strategies to reduce the cost of preventive and restorative maintenance, while improving the level of equipment reliability and service life, reducing the number of repairs, and realising the dual goals of cost savings and safety enhancement.

Railroad locomotives operate in complex and changing operating environments, and key components such as High Voltage Electrical Apparatus, Traction Motors, Bogies, etc. are prone to defects or failures and require efficient overhaul and maintenance. The current maintenance system relies on fixed periodic maintenance, there are problems such as over maintenance, accessories delivery and repair time is not stable, high maintenance cost, long time-consuming and difficult to ensure the quality of maintenance, etc. Reasonable repair schedule and repair system can guarantee the safe operation of harmonious locomotives, and at the same time, reduce the cost of maintenance and improve efficiency.

### Literature review

In recent years, equipment maintenance strategies and production process optimisation have become a research hotspot in academia and engineering. In the field of single component systems, Zhao F et al. [1] proposed a preventive maintenance framework for general production systems by considering the maintenance problems of production systems with maintenance delays and accessibility to dynamic environments. Ali M B et al. [2] established a condition-based preventive maintenance strategy by considering reliability as a key constraint. Xiaoliang H et al. [3] introduced an incomplete preventive maintenance (IPM) mechanism, which was combined with a hybrid failure rate model to quantify maintenance effectiveness. For the railway field, Rudek R et al. [4] proposed an optimization model for preventive maintenance of railroad vehicles based on heuristic and meta-heuristic algorithms. Cheng et al. [5] combined incomplete preventive maintenance with a minimum maintenance strategy to establish an extended warranty model for multi-component systems with the goal of maximizing availability. Yang et al. [6] proposed a strategy for optimising the maintenance costs of electric locomotives from a life-cycle cost perspective.

In the study of multicomponent complex systems, Shen et al. [7] introduced the buffer inventory setting and service age regression theory to optimise maintenance schedules. Herguedas et al. [8] determined optimal engine maintenance intervals using a semi-Markov process. Elleuch et al. [9] developed a system for predicting the deterioration of track geometry. M. Szkoda et al. [10] compared preventive and aftercare strategies through fault tree analysis. Lin.J et al. [11] supported machine wheel-to-maintenance decisions through Bayesian degeneration analysis. Rui et al. [12] optimised wind turbine maintenance strategies with a semi-Markov decision process. Mohammed et al. [13] calculated the reliability of the unloading system for cargo vehicles based on failure rate. Sheng Lin et al. [14] proposed a preventive opportunity maintenance (POM) approach based on equipment reliability for railway traction power supply systems (TPSS). The method modeled the equipment degradation process using a Weibull distribution and optimises maintenance programs to minimise total outage time, proven to significantly reduce unplanned outage time and improve efficiency. Binder M et al. [15] systematically assessed the potential of predictive maintenance algorithms for railway applications.Hu et al. [16] dynamically adjusted the failure rate model by means of a service age regression factor. Zhu et al. [17] constructed a preventive maintenance model based on normal distribution degradation.

Despite significant progress in existing research, limitations in maintenance strategies for multi-component systems remain. For one thing, most models assume that components operate independently, ignoring the interactions and synergies between them. Secondly, fixed-cycle maintenance tends to lead to over repairs. Third, insufficient capacity for dynamic adjustment of complex systems. This paper proposes a maintenance optimisation model for multi-component systems that addresses the shortcomings of the traditional model by dynamically adjusting maintenance schedules to achieve an overall improvement in cost, efficiency and quality. The novelty of this paper lies in the fact that, for the first time, a chance grouping strategy combined with a genetic algorithm that apply to maintenance optimisation of multi-component locomotive systems. It also introduces a service age regression factor and a failure rate increment factor to dynamically adjust the maintenance cycle. It solved the pain point of over-maintenance in traditional fixed-cycle maintenance.

### Harmony locomotive C4 repair status and problems

Railroad locomotive repair system is an important guarantee to ensure the safe and reliable operation of locomotives, which stipulates the maintenance work that should be carried out in different running mileage or time intervals. According to the use of locomotives and the wear and tear of parts, the repair system is usually divided into six repair levels from C1 to C6, of which C4 repair is particularly critical [18]. C4 repair plays the role of acting as a bridge between what precedes and what follows in the reform of repairing system, lays the foundation for the subsequent C5 and C6 high-level repairing, and effectively reduces the maintenance cost and workload of the subsequent high-level repairing. This paper provides an in-depth study of the C4 repair preventive maintenance strategy for harmonious electric locomotives.C4 repair, as a high level of segmental repair program, has a cycle of 500,000 kilometers or not more than 3 years. The content of C4 repair includes disassembling, inspecting, repairing or replacing the main parts of locomotives such as traction motors, main transformers, bogies, car bodies, etc., and carrying out a comprehensive test and adjustment of locomotive’s electrical system, braking system and control system. Through C4 repair, it can effectively eliminate the hidden faults of the locomotive, restore its performance, prolong its service life, and ensure the safety and reliability of the locomotive in the subsequent operation.

### Harmony locomotive C4 repair general operating procedure

**Fig 1:**
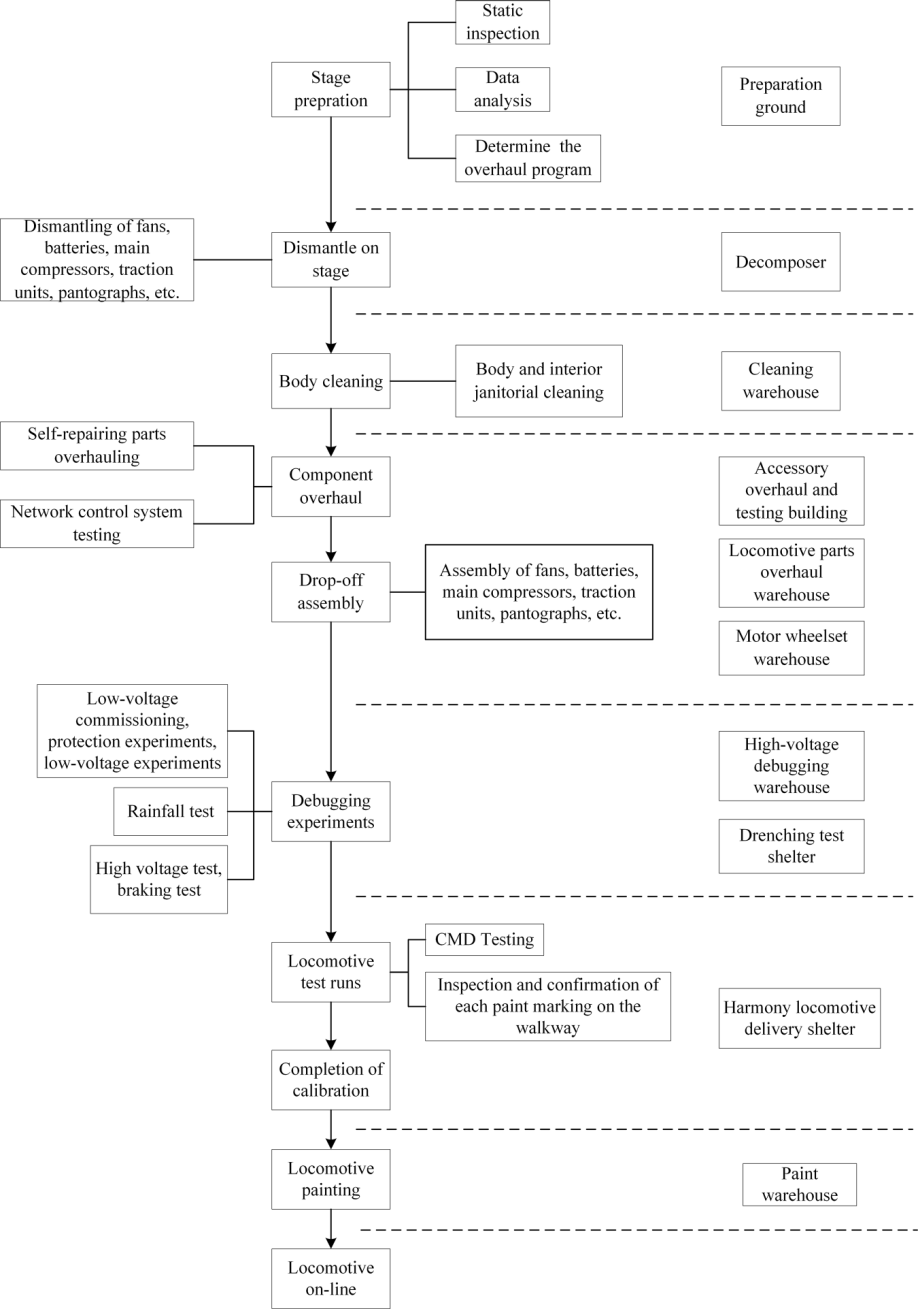
C4 repair operation flow chart.

Harmony electric locomotive C4 repair operations in the general process mainly includes pre-stage preparation, dismantle on stage, car body cleaning, component overhaul, drop-off assembly, debugging experiments, locomotive test run, completing of calibration, locomotive painting and locomotive on-line of these ten steps, as shown in Fig 1. For some models and some maintenance links, due to the lack of necessary maintenance equipment or maintenance materials in some smaller sections, locomotive parts will be disassembled and transferred to other sections or technical companies for external repair, and then returned to this section to be assembled for subsequent maintenance processes.

Involved in the plant generally include C4 repair warehouse, cleaning warehouse, accessory overhaul warehouse, motor wheelset warehouse, high-voltage debugging warehouse, central spare parts warehouse, turnbuckle warehouse, paint warehouse, accessory overhaul and testing building, harmonious accessory building, delivery shelter, drenching test shelter and so on.

### Analysis of existing problems

**Fig 2:**
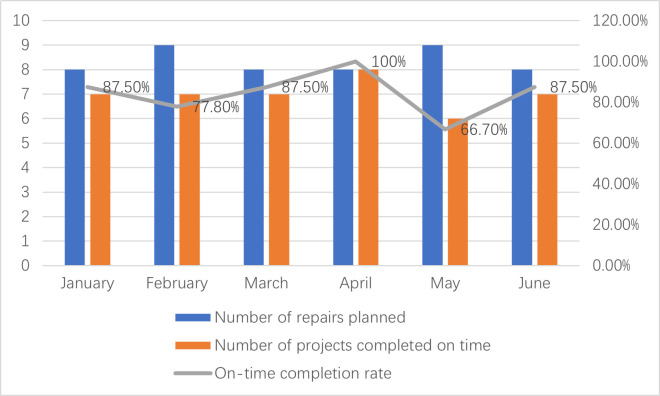
On-time completion rate of C4 repairs in the first half of 2023 at Y Locomotive Depot.

Y Locomotive Depot is one of the first units in China to be qualified for C4 repair and maintenance of harmonious electric locomotives. It is also the largest C4 repair and maintenance depot in the group of railway bureaus where it is located. In this paper, we take the Y Locomotive Depot as an example, according to the data collected from on-site research, it is known that in the first half of 2023, the C4 repair of the section was completed for a total of 50 units, and 8 locomotives were overhauled and shut down, and the months of overhauling were as shown in Fig 2. In February and May, two locomotives were sent out for repair.

**Fig 3:**
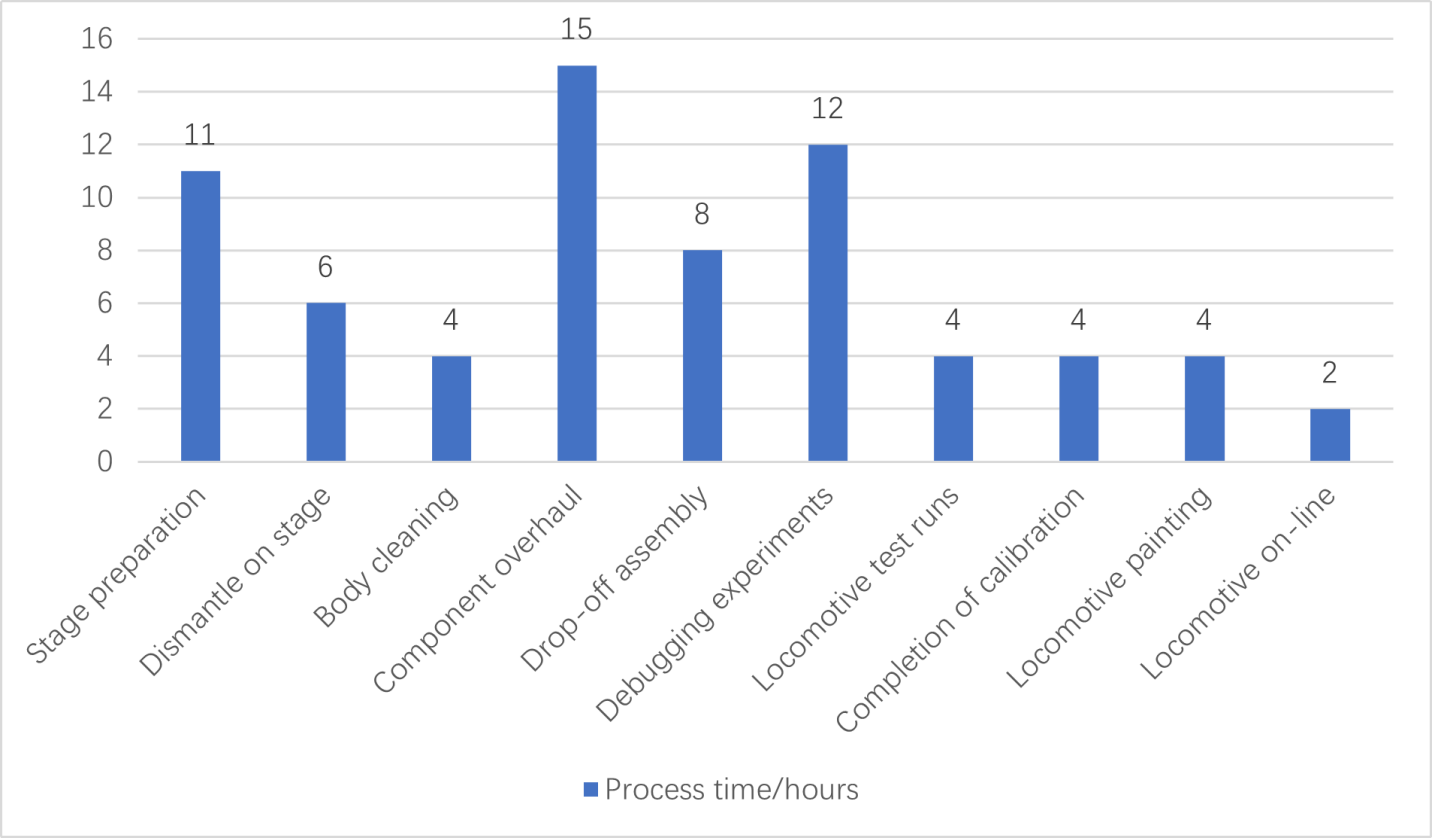
C4 repair operation time for each step of the process.

The time taken for each part of the C4 repair operation is shown in Fig 3 (from S1 File), which shows that the three links of stage preparation, component overhaul and debugging experiments account for the most relative time in the whole C4 repair process, totaling 38 hours, accounting for 54.3% of the whole.

According to Table 1 of the partially completed locomotive stopping time situation in the quality analysis report of mid-repair in the first half of 2023 of Y Locomotive Depot, locomotives are more prone to overhaul stopping time overruns when they are commissioned and sent for repair.

**Table 1:** Table of completed locomotive stoppages in the first half of 2023.

Serial number	Locomotive models	Date of departure from the Locomotive Depot	Date of return to the Locomotive Depot	Return depot refurbishment Downtime/hours	Total stopping time/hour	Main reasons for overtime for refurbishment
1	DF7C	23-2-10	23-4-18	11.0	69.0	Anti-relaxation mainframe without power
2	DF7C	23-3-12	23-5-16	14.0	65.0	Audio and video malfunctions
3	DF7C	23-4-29	23-6-18	10.0	50.0	Cooling fan automatic bit jammed
4	DF4B	23-4-15	23-6-14	16.0	60.0	Right 2, right 3 brake cleat installation is not standardized
5	DF7C	23-5-15	23-6-15	19.0	77.0	Failure of wind pump
6	DF4B	23-4-10	23-6-14	13.0	66.0	/

There are still some problems in the current harmonious locomotive C4 repair operation, which directly affect the efficiency and quality of maintenance. First of all, the current overhaul of China’s railroad locomotives mainly adopts the approach of multi-level and equal cycle. Although this kind of overhaul can basically ensure the stable operation of the equipment, due to the different functions borne by different parts in the body, the losses occurring in the actual operation are also different. Therefore, if the maintenance program adopts a consistent overhaul cycle, the probability of random failure of components will be greatly increased.

Secondly, the current maintenance scope of locomotive components generally covers more than 10 parts, including traction motors and auxiliary units. Certain locomotive components with a low utilization or failure rate will be repeatedly disassembled and inspected in multi-level maintenance, which is very easy to produce excessive maintenance problems and a serious waste of manpower and time resources. Excessive maintenance also generates a lot of additional operational requirements, resulting in consistently high locomotive maintenance costs.

In addition, the intermediate repair and overhaul of the harmonious locomotive often exists in the case of joint repair of multiple repair depot, due to the outsourcing of parts to send repair time-consuming can not be accurately controlled, so that the maintenance cycle of the vehicle is not stable enough, and if due to a variety of reasons so that the parts are delayed in the round-trip process will be even more reduce the efficiency of the repair, and disrupt the subsequent train maintenance program.

The existence of these problems makes the locomotive maintenance costs remain high, maintenance efficiency is low, and maintenance quality is difficult to be guaranteed.

### Single component inspection cycle optimization strategy

This paper first takes single component system as the research object, determines the initial state of the locomotive and maintenance key components according to the actual situation and the degree of fault hazard, and establishes the single component inspection cycle optimization model with the degree of reliability as the constraint under the optimal maintenance cost. Then, considering the influence of the age reduction factor and the failure rate increase factor on the failure rate of components, the single component elastic preventive maintenance cycle is determined.

In order to make the modeling more relevant, the paper makes the following assumptions:

1) The occurrence of defects in the components of the system will not affect each other, and the incidence of defects is recorded as *λ*. In complex systems similar to railroad locomotive systems, the occurrence of component defects usually obeys a Poisson process.
2) System components go through normal and defective phases before failing. Denote the defective phase time according to the previous section as *h*, its probability density function as *f(h)*, and the distribution function as *F(h)*;
3) Only one failure of a system component can occur at any one time;
4) System components will be in new condition after preventive maintenance. The above three assumptions are made to facilitate the construction of a detection cycle model based on delay time theory.
5) Preventive maintenance activities do not fully restore the state of the system components and the probability of defects *r* exists 0<r<1 ;
6) If a locomotive breaks down during operation it does not stop immediately, but continues to run to the nearest locomotive section where the breakdown is repaired. The above two assumptions are made to make the model more consistent with the actual utilization of the locomotive.

### Single component inspection cycle optimization model

Assume that the probability that a component is defective at moment *u* fails at moment p(t|u). Find the failure repair cost of the component.

The probability that a defect in a component at moment *u* is detected at moment *iT* is p(iT|u). Find the cost of preventive maintenance. Add the two repair costs to obtain the total repair cost per unit time of the locomotive component as:


$$\begin{array}{c} Min\overset{―}{C}(T)=\frac{\int_{0}^{T}\left(\sum_{n=1}^{t}\left(\left(i-n+1\right)c_{s}+c_{f}\right)\times p\left(t|u\right)\right)dt}{T}\\ \;\;\;\;\;+\frac{\int_{0}^{\infty }\left(\left(i-n+1\right)c_{s}+c_{r}\right)\times p\left(iT|u\right)du}{T} \end{array}$$ (1)

In the formula: *c(t)* is the expected value of losses due to faulty repairs at moment t((i−1)T<t<iT) *c*_*s*_ is the cost of detecting the defect; *c*_*r*_ is the cost of repairing the defect; *c*_*f*_ is the cost of fault repair; *c*_*iT*_ is the expected value of losses due to preventive maintenance at *iT* time;

When the preventive maintenance activity is carried out for the times of *i*, *n*_*i*_ *(i = *1,2*,…,n)* defects are detected, at which point the preventive inspection cycle is divided into *k* mutually independent intervals of length Δt, which are expressed as:


$$I_{j}^{i}=\left[\left(i-1\right)T+\left(j-1\right)\Delta t,\left(i-1\right)T+j\Delta t\mathrm{\backslash rightleft}(j=1,2,...,k\right)$$ (2)

In the formula: *T* is the preventive maintenance cycle; *iT* is the moment of the *i* preventive maintenance.

Since the occurrence of a defect in a component and the development of a defect into a failure are independent of each other are not correlated. Therefore the estimated parameter is the product of the probabilities of these two events:


$$L=\prod_{i=1}^{n}\left\{P\left(\text{number of defects found at iT}{\text{n}}_{\text{i}}\right)\prod_{j=1}^{k}P\left({\text{number of failures occurring in interval I}}_{\text{j}}^{\text{i}}{\text{m}}_{\text{ij}}\right)\right\}$$ (3)

In the formula: *n*_*i*_ is the number of defects detected at *iT* moment; *m*_*ij*_ is the number of faults occurring on $I_{j}^{i}$.

**Fig 4:**
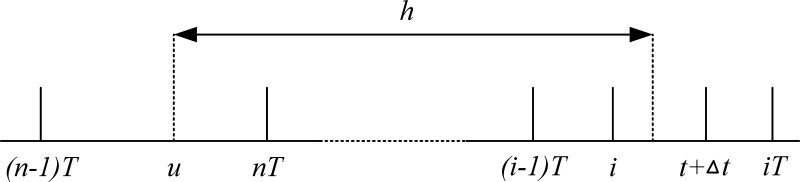
Defect deterioration failure diagram.


Assume that the probability that a defect occurs in a component at moment *u* and fails at moment *t* is p(t|u), as shown in Fig 4. where (i−1)T<t<iT.

$$p\left(t|u\right)=\left\{ \begin{array}{c} {\left(1-r\right)}^{i-n+1}\left(\begin{array}{c} F\left(t-\left(n-1\right)T\right)\\ -F\left(t-nT\right) \end{array}\right)\left(n-1\right)T<u<nT,n=1,2,...,i-1\\ F\left(t-\left(i-1\right)T\right)\;\;\;\;\;\;\;\;\;\;\left(i-1\right)T<u<iT\\ 0\;\;\;\;\;\;\;\;\;\;\;\;\;\;\;\;other \end{array}\right.$$ (4)

Where *r* denotes the repair rate, which represents the probability that a defect is repaired after each inspection(0≤r≤1). *T* denotes locomotive operating cycle. *F* denotes the Cumulative Distribution Function (CDF), which describes the distribution of defects from the time they occur to the time they are transformed into failures.

The average number of failures occurring in $I_{j}^{i}$ is:


$$\begin{array}{c} EN_{f}\left(I_{j}^{i}\right)=\int_{\left(i-1\right)T}^{iT}v\left(t\right)dt\\ \;\;\;\;\;\;=\int_{\left(i-1\right)T}^{iT}\left\{\begin{array}{c} \sum_{n=1}^{i}{\left(1-r\right)}^{i-n+1}\left[F\left(t-\left(n-1\right)T\right)-F\left(t-nT\right)\right]\\ +F\left(t-\left(i-1\right)T\right) \end{array}\right\}dt \end{array}$$ (5)

Then the probability of generating *m*_*ij*_ faults during$I_{j}^{i}$ is:


$$P\left(\text{number of faults occurring in interval }I_{j}^{i}m_{ij}\right)=\frac{{\left[EN_{f}\left(I_{j}^{i}\right)\right]}^{m_{ij}}e^{-EN_{f}I_{j}^{i}}}{m_{ij}!}$$ (6)

Assume that a defect occurs at moment *u* and is detected at moment *iT* with probability p(iT|u).


$$p\left(iT|u\right)=\left\{ \begin{array}{c} {\left(1-r\right)}^{i-n}r\left(1-F\left(iT-u\right)\right)\left(n-1\right)T<u<nT,n=1,2,...,i-1\\ r\left(1-F\left(iT-u\right)\right)\;\;\;\;\left(i-1\right)T<u<iT\\ 0\;\;\;\;\;\;\;\;\;\;\;\;other \end{array}\right.$$ (7)

where ${\left(1-r\right)}^{i-n}$ denotes the probability that the defect is not repaired in the subsequent i−n tests. r(1−F(iT−u)) denotes the probability of being repaired in the current inspection multiplied by the probability that the defect has not been converted into a fault.

Then the expected value $EN_{p}\left(iT\right)$ of defects occurring when the number of preventive tests reaches *i* is:


$$\begin{array}{c} EN_{p}\left(iT\right)=\lambda \int_{0}^{\infty }P\left(iT|u\right)dx=\lambda \sum_{n=1}^{i}{\left(1-r\right)}^{i-n+1}r\int_{\left(n-1\right)T}^{nT}\left[1-F\left(iT-u\right)\right]du\\ \;\;\;\;\;+\lambda r\int_{\left(i-1\right)T}^{iT}\left[1-F\left(iT-u\right)\right]du \end{array}$$ (8)

Based on the assumptions above, the failure of a system component is a random event and follows a Poisson distribution. Therefore, the probability that the number of defects detected at *iT* moment is *n*_*i*_ is:


$$P\left(number\;of\;defects\;found\;at\;i\;T\;moment\;n_{i}\right)=\frac{{\left[EN_{p}\left(iT\right)\right]}^{n_{i}}e^{-EN_{p}\left(iT\right)}}{n_{i}!}$$ (9)

Bringing Equations (2.6) and (2.9) into Equation (2.3) yields:


$$\ln L=\sum_{i=1}^{n}\left(n_{i}\ln EN_{p}\left(iT\right)-EN_{p}\left(iT\right)\right)+\sum_{i=1}^{n}\sum_{j=1}^{k}\left(m_{ij}\ln EN_{f}\left(I_{j}^{i}\right)\right)$$ (10)

The parameters in the delay time distribution function are brought into the preventive detection model, and each different value of the detection cycle is taken to correspond to a different repair cost, and an image of the change in the function values of the two is obtained. The optimal inspection cycle with the smallest repair cost is determined based on the image variation.

### Single-component flexible service intervals

The condition of a locomotive component cannot usually be restored to a new condition after a repair operation. According to the delay-time theory, components go through the process of moving from the normal phase to the delay time phase and finally to the failure phase, and ideally, defects in locomotive components are detected and preventive maintenance is carried out as soon as they occur. Therefore, by considering the age reduction factor and the failure rate increase factor in the optimal inspection cycle, each repair operation will bring the component back to the new one, but the probability of occurrence of defects will increase to shorten the optimal inspection cycle. In order to ensure the reliability of the component it is necessary to gradually increase the number of repairs. In practice, preventive maintenance does not completely restore the state of a component to a new state, i.e., incomplete maintenance.

### Age reduction factor

Indicates the extent to which the performance of a system component is restored after an incomplete repair operation, denoted as *α*_*i*_, and the working life after repair is referred to as the effective service life.


$$\alpha_{i}={\left(a\times \frac{C_{pm_{i}}}{C_{pr}}\right)}^{bi},i=1,2,...,N-1$$ (11)

Where *a* indicates the improvement parameter of system or component maintenance cost, reflecting the recovery effect of preventive maintenance of different maintenance costs on the system or component, and *b* is the adjustment parameter of the number of times preventive maintenance is carried out, in which $1\leq a\leq \left(C_{pr}/C_{pm_{i}}\right)$, 0<b<1. The values of *a* and *b* can be obtained by counting the historical failure data of the system or component; $C_{pm_{i}}$ denotes the cost of a single incomplete preventive maintenance; $C_{pr}$ denotes the cost of a single preventive replacement operation of the component, where $C_{pm_{i}}<C_{pr}$ *N* denotes the number of preventive maintenance.

### Failure rate increase factor

Denote that the failure rate function increases by a factor of *β*_*i*_ after a system component undergoes an incomplete repair operation, although the failure rate is zeroed out. This situation can therefore be represented by using the failure rate increment therefore, as specified by the rule:


$$\lambda_{k}\left(t\right)=\beta_{k-1}\lambda_{k-1}\left(t\right)=\left(\prod_{i=1}^{k-1}\beta_{i}\right)\lambda \left(t\right),1<\beta_{1}\leq \beta_{2}\leq ...\leq \beta_{k-1}$$ (12)

In practical work and maintenance activities, the effective working life of a system component decreases as the number of repairs increases, while the failure rate function gradually increases. Therefore the combination of two maintenance improvement factors establishes a hybrid failure rate function:


$$\lambda_{i+1}\left(t\right)=\left(\prod_{k=1}^{i}\beta_{k}\right)\lambda \left(\sum_{k=1}^{i}\alpha_{k}T_{k}+t\right),t\in \left(0,T_{i+1}\right)$$ (13)

where the values of *α*_*k*_ and *β*_*k*_ can be obtained from previous maintenance records.

### Delay time function determination

The Weibull distribution, because of its remarkable flexibility in adapting to various shapes of distribution curves, is uniquely suited to characterize the relationship between key quantities such as component defect probability, operating life, and delay time. This property of the Weibull distribution makes it particularly important in the reliability analysis of complex systems, as it is able to accurately represent the intrinsic relationship between these quantities without the need to specify a trend. Therefore, given its strong adaptability and accuracy, this paper chooses to use the Weibull distribution to analyze the delay time.

**Fig 5:**
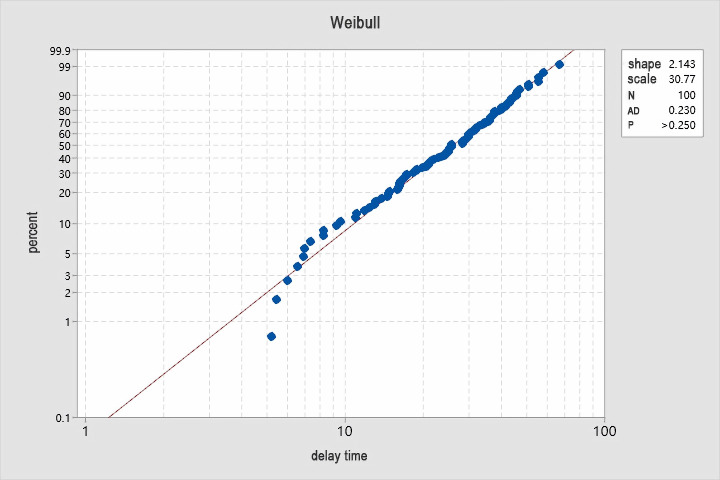
Delay time probability.

The increase in the probability of a defective part decreases the working life with respect to the delay time stage. Assuming that the delay time *h* obeys a certain random variable, noting that the probability function is *f(t)* and the distribution function is *F(t)*. Using Minitab software to select a key component of the locomotive to analyze its historical maintenance data with AD statistics, a probability distribution graph is obtained as shown in Fig 5.

From the above figure it can be seen that the p-value is greater than 0.250 and the delay time of this system component obeys a Weibull distribution and hence the failure rate function is:


$$\lambda_{a}=\left(\frac{\delta_{a}}{\eta_{a}}\right){\left(\frac{t}{\eta_{a}}\right)}^{\delta_{a}-1}$$ (14)

Where: $\delta_{a}$ denotes the shape parameter; $\eta_{a}$ denotes the scale parameter.

Their values can be obtained from the locomotive component maintenance history using the great likelihood estimation method.

**Fig 6:**
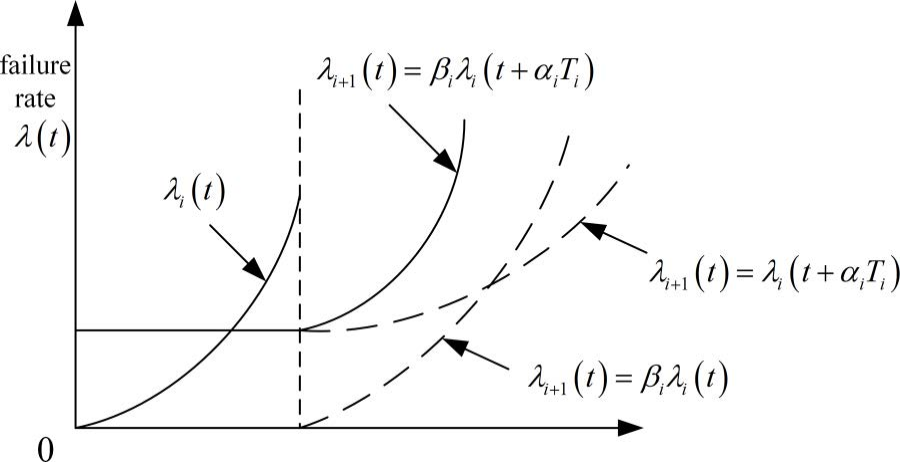
Delay time failure rate evolution model.

The Improvement Factor [19] can show the effect of reliability improvement of locomotive components after each preventive maintenance, with the following trend: the failure rate of a locomotive component will not be cleared after preventive maintenance operations but will be at the value of the normal phase at a certain moment, but the rate of increase of the failure rate will increase. The next preventive maintenance will come sooner, i.e., the maintenance will become more frequent. The variation of the failure rate *λ(t)* is shown in Fig 6.

Thus the failure rate curve function considering the maintenance improvement factor is:


$$\lambda_{i+1}\left(t\right)=\beta_{i}\lambda_{i}\left(t+\alpha_{i}T_{i}\right),t\in \left(0,T_{i+1}\right)$$ (15)

The values of *α*_*i*_ and *β*_*i*_ in Eq. can be obtained from the maintenance history of locomotive components using the maintenance cycle fitting method. In order to simplify the subsequent calculation process can be made $\alpha_{i}=\alpha ,\beta_{i}=\beta$.

A component *a* is repaired when its reliability is equal to its minimum reliability threshold $R_{am}$. The reliability equation is:


$$\int_{0}^{T_{Y1a}}\lambda_{Y1a}\left(t\right)dt=\int_{0}^{T_{Y2a}}\lambda_{Y2a}\left(t\right)dt=...=\int_{0}^{T_{Yia}}\lambda_{Yia}\left(t\right)dt=-\ln R_{am}$$ (16)

The preventive maintenance cycle for locomotive components *a* is the optimal inspection cycle plus a delay time of:


$$T_{ia}=T_{Jia}+T_{Yia}$$ (17)

The maintenance cost per unit of time over the operating cycle of part *a* is:


$$\overset{―}{C_{a}}=\frac{\sum_{i=1}^{N_{a}}\left[\left(C_{fa}\left(-\ln R_{am}\right)\right)+C_{pa}+C_{sa}+C_{da}\left(\tau_{pa}+\tau_{fa}\left(-\ln R_{am}\right)\right)\right]+C_{ra}}{\sum_{i=1}^{N_{a}}\left[T_{Yia}+\tau_{pa}+\tau_{fa}\left(-\ln R_{am}\right)\right]+\tau_{ra}}$$ (18)

The optimal number of preventive maintenance in the working life cycle *N*_*a*_ is obtained by solving for the minimum value of maintenance cost per unit time of component *a*. The final optimization model of preventive maintenance schedule is:


$$\left\{ \begin{array}{c} \min{\overset{―}{C}}_{a}=\frac{\sum_{i=1}^{N_{a}}\left[\left(C_{fa}\left(-\ln R_{am}\right)\right)+C_{pa}+C_{sa}+C_{da}\left(\tau_{pa}+\tau_{fa}\left(-\ln R_{am}\right)\right)\right]+C_{ra}}{\sum_{i=1}^{N_{a}}\left[T_{Yia}+\tau_{pa}+\tau_{fa}\left(-\ln R_{am}\right)\right]+\tau_{ra}}\\ T_{ia}=T_{Jia}+T_{Yia}\\ \int_{0}^{T_{Yia}}\lambda_{Yia}\left(t\right)dt=-\ln R_{am}\\ R_{am}>0;N_{a}>0;i=1,2,...,N_{a},T_{Yia}>0 \end{array}\right.$$ (19)

### Multi-component scheduling preventive maintenance plan optimization strategy

By virtue of its high degree of specificity, single-component maintenance can focus on the detailed maintenance and management of specific components. However, considering that there is a non-negligible mutual influence and synergy between components, in order to more comprehensively improve the maintenance efficiency and system reliability, this paper further investigates the optimization model of the multi-component maintenance plan, in order to comprehensively consider the correlation between the components, and realize the efficient maintenance of the whole system.

**Fig 7:**
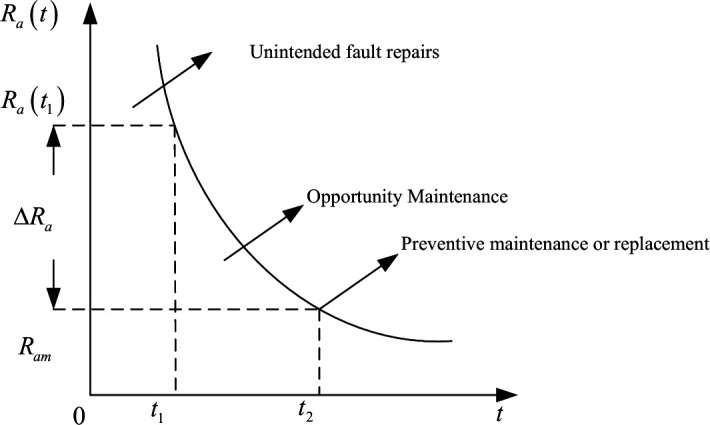
Opportunity group maintenance strategy.

When the locomotive system adopts a group maintenance strategy, multiple components are jointly maintained at every point in time T,2T,...,nT(n∈N), where *T* is the minimum maintenance cycle. However, in actual component operating conditions, failures do not all occur at the same time, preventing a better group maintenance program. Therefore, this paper combines group maintenance with opportunity maintenance, when a component in the system reaches the optimal maintenance cycle, determine whether the reliability of all components is lower than the set reliability threshold, and group the components that do not meet the standard for maintenance, while the rest of the components are not subject to preventive maintenance. The maintenance strategy is shown in Fig 7. The plot curve theoretically represents the process of decreasing the reliability threshold of a component within a system over time, with the component operating time *t* as a function of its reliability $R_{a}\left(t\right)$ depending on the specific object of study.

1) If $R_{a}\left(t\right)-R_{am}>\Delta R_{a}$, then no opportunistic group repair is made to part *a* at time *t*;
2) If $0<R_{a}\left(t\right)-R_{am}\leq \Delta R_{a}$, then opportunistic group repair of component *a* is performed at time *t*;
3) If $R_{a}-R_{am}\leq 0$, then preventive replacement of part *a* is carried out.

Too high or too low a minimum reliability threshold $\Delta R_{am}$ can cause an increase in the total maintenance cost, and it is necessary to find an appropriate reliability threshold to reduce the total maintenance cost of the locomotive system during the operating cycle while ensuring the safe operation of the railroad locomotive. The direct maintenance cost of Component *a* due to maintenance operations during two adjacent preventive maintenance intervals is:


$$C_{pan}=\left\{ \begin{array}{cc} 0 & {\text{S}}_{{at}_{n}}=N\\ C_{pa} & {\text{S}}_{{at}_{n}}=M\\ C_{ra} & {\text{S}}_{{at}_{n}}=R\\ C_{fa}\cdot \int_{n-1}^{n}\lambda_{Yia}(t)dt & \text{unintended fault repair} \end{array}\right.$$ (20)

Where $S_{at_{n}}$ represents the maintenance strategy adopted by component *a* in different cases. *N* means that component *a* does not undergo any maintenance operation; *M* means that component *a* undergoes preventive maintenance; and *R* means that component *a* undergoes preventive replacement.

The direct cost of component *a* is expressed as:


$$C_{a}=\sum_{n=1}^{N}C_{pan}$$ (21)

Thus the direct maintenance cost of the system is expressed as:


$$C_{x}=\sum_{a=1}^{M}\sum_{n=1}^{N}C_{pan}$$ (22)

Generally, the longer the downtime, the more money is lost. The locomotive system is a tandem system, and the locomotive cannot operate without any of its components, so the locomotive downtime is equal to the component repair time. The loss of the locomotive system due to downtime is then:


$$C_{pd}=C_{d}T_{pd}=C_{d}\sum_{n=1}^{N}T_{pdn}$$ (23)

Number of preventive maintenance for component *a* during cycle *T*:


$$m_{a}=pN_{a}+q\left(p,q=0,1,...\right)$$ (24)

Where: *p* is the number of replacement repairs; *q* is the number of preventive maintenance sessions remaining after replacement maintenance.

Then the downtime of the locomotive due to failure is:


$$T_{fda}=\tau_{fa}\left[p\sum_{j=1}^{N_{a}}\int_{0}^{T_{Yja}}\lambda_{Yja}\left(t\right)dt+\sum_{j=1}^{q}\int_{0}^{T_{Yja}}\lambda_{Yja}\left(t\right)dt\right]$$ (25)

The system spends on downtime due to faulty repairs in cycle *T* is:


$$C_{fd}=C_{d}T_{fd}$$ (26)

Associating Equations (22), (23), and (26), the total maintenance spend of the system over the operating cycle *T* is:


$$\begin{array}{c} C=C_{x}+C_{pd}+C_{fd}\\ \;=\sum_{a=1}^{M}\sum_{n=1}^{N}C_{pan}+C_{d}\sum_{n=1}^{N}T_{pdn}\\ \;+C_{d}\sum_{a=1}^{M}\tau_{fa}\left[p\sum_{j=1}^{N_{a}}\int_{0}^{T_{Yja}}\lambda_{Yja}\left(t\right)dt+\sum_{j=1}^{q}\int_{0}^{T_{Yja}}\lambda_{Yja}\left(t\right)dt\right] \end{array}$$ (27)

Locomotive system availability is the percentage of uptime over the entire operating cycle. If the uptime is too short, it will be detrimental to the utilization of the locomotive and thus reduce the revenue efficiency of the railroad line. Therefore, in order to efficiently utilize line resources and reduce unnecessary maintenance costs, it is necessary to maximize locomotive system availability. In order to reduce the influence of the extremely large and small values of the availability on the model, this paper calculates the use of the availability of the average availability. A certain value exists when the preset reliability threshold increases from small to large, at which time the corresponding locomotive system downtime is minimized, i.e., the availability is maximized, which is expressed as:


$$\begin{array}{c} A=\frac{U}{U+D}=1-\frac{D}{U+D}\\ \;\;\;\;\;\;=1-\frac{\sum_{n=1}^{N}T_{pdn}+\sum_{a=1}^{M}\tau_{fa}\left[p\sum_{j=1}^{N_{a}}\int_{0}^{T_{Yja}}\lambda_{Yja}\left(t\right)dt+p\sum_{j=1}^{q}\int_{0}^{T_{Yja}}\lambda_{Yja}\left(t\right)dt\right]}{T} \end{array}\;$$ (28)

Where: *U* is the system uptime; *D* is the system downtime.

Then the maintenance plan optimization model with the objective of maximum availability can be expressed as:


$$\left\{ \begin{array}{c} \text{max}A\left(\overset{⇀}{\Delta R}\right)=1-\frac{\sum_{n=1}^{N}T_{pdn}+\sum_{a=1}^{M}\tau_{fa}\left[p\sum_{j=1}^{N_{a}}\int_{0}^{T_{Y}ja}\lambda_{Yja}(t)dt+p\sum_{j=1}^{q}\int_{0}^{T_{Yja}}\lambda_{Yja}(t)dt\right]}{T}\\ s.t\;-\ln\left(R_{am}+\Delta R_{a}\right)\leq \int_{0}^{T_{Yia}}\lambda_{Yia}(t)dt\leq -\ln R_{am}\\ 0\leq \Delta R_{a}\leq \min(1-R_{am}) \end{array}\right.$$ (29)

### Model solving algorithm design

Traditional algorithms usually use linear programming, dynamic programming and other methods to solve the preventive maintenance plan optimisation model for multi-component opportunity grouping. These methods are more effective in dealing with simple optimization problems, but have limitations such as high computational complexity, easy to fall into local optimal solutions, and poor adaptability when faced with complex multi-component systems. And genetic algorithm is a search algorithm based on the principles of natural selection and genetics, which has a strong global search capability, is suitable for high-dimensional non-linear optimisation problems, and can effectively avoid local optimal solutions. Since locomotive maintenance involves multi-component co-optimisation and the objective function contains high-dimensional nonlinear constraints, the paper introduces a heuristic genetic algorithm that can speed up the convergence of the model solution and find the optimal solution accurately and efficiently.

The steps for solving the optimization model of preventive maintenance plan for multi-component schedules are as follows:

1) Collecting past maintenance records from the maintenance section and analyzing and solving the network data to obtain the relevant parameters $C_{fa}$, $C_{pa}$, $C_{da}$, $C_{ra}$, $\tau_{pa}$,$\tau_{fa}$, $\tau_{ra}$, $R_{am}$, $\alpha_{ia}$, $\beta_{ia}$, $\delta_{a}$, $\eta_{a}$ and the distribution function of the delay time of each key component. Then the delay time $T_{Yia}$ of the critical component *a* under the minimum maintenance cost is found according to Equation (18). From Equation (17), the optimal preventive maintenance cycle $T_{ia}$ is obtained by adding the delay time $T_{Yia}$ with the optimal preventive inspection cycle $T_{Jia}$.
2) Calculate the moment $t_{n}=\min\left(t_{1n},t_{2n},...,t_{an},...,t_{Mn}\right)$ of the *n*th preventive maintenance of the locomotive system.
3) Compare the magnitude of the difference between the current reliability and the minimum reliability, $R_{an}-R_{am}$, in relation to the preset reliability threshold, $\Delta R_{a}$. When $R_{an}-R_{am}>\Delta R_{a}$, the reliability of component *a* is high at time *t*_*n*_ and no maintenance operation is needed; when $0<R_{an}-R_{am}\leq \Delta R_{a}$, the reliability of component *a* decreases at time *t*_*n*_, and it is necessary to carry out preventive maintenance for the opportunity group to ensure the safety of locomotive operation; If $0<R_{an}-R_{am}\leq \Delta R_{a}$ and $n_{a}=N_{a}+1$, component *a* does not meet the basic requirements for locomotive operation in terms of reliability at time *t*_*n*_, it is preventively replaced in groups of opportunities, and the number of preventive repairs *n*_*a*_ for the newly replaced component *a* is recalculated.

The preventive maintenance time for component *a* at time *t*_*n*_ is:


$$\left\{ \begin{array}{c} \tau_{pat_{n}}\;\;\;\;\;\;\;\;\;S_{at_{n}}=M\\ \tau_{rat_{n}}\;\;\;\;\;\;\;\;\;S_{at_{n}}=R\\ 0\;\;\;\;\;\;\;\;\;\;\;\;\;\;\;\;\;\;S_{at_{n}}=N \end{array}\right.$$ (30)

Where: $\tau_{pat_{n}}$ denotes the time spent on preventive maintenance of component *a* at time *t*_*n*_, and $\tau_{rat_{n}}$ denotes the time spent on preventive replacement of component *a* at time *t*_*n*_. After n−1 preventive maintenance of the locomotive system due to the maintenance improvement factor, the *n*th maintenance is the most difficult to operate and should have the longest downtime, i.e., $T_{pdn}=\max\left(\tau_{pat_{n}},\tau_{rat_{n}}\right)$.

4) From Equation (22), find the direct cost *C*_*x*_ incurred by the maintenance operation of component *a* during the period of time when the *n*th preventive maintenance is completed; and from Equations (23) and (26), find the expenses incurred by the locomotive system due to the shutdown of the locomotive system for the preventive maintenance and the breakdown maintenance. The total maintenance cost of the *n*th preventive maintenance of the locomotive system is found from Equation (27).
5) The moment when critical component *a* undergoes its n+1 st repair is:

$$t_{a\left(n+1\right)}=\left\{ \begin{array}{c} t_{an}+T_{dn}\;\;\;\;\;\;\;\;\;\;\;\;\;\;\;\;\;\;\;\;\;\;\;\;\;\;\;\;\;\;\;S_{at_{n}}=0\\ t_{n}+T_{a\left(n_{a}+1\right)}+T_{dn}\;\;\;\;\;\;\;S_{at_{n}}=M,R \end{array}\right.$$ (31)

Repeat steps 2–4 until $t_{n}=N+1>T$. From Equation (27), the maintenance cost derived each time is brought in to obtain the total maintenance cost of the locomotive system over its specified operating life. Then calculate the availability *A* of the locomotive system from Equation (28).

For the setting of the optimal reliability threshold $\vec{\Delta R_{A}^{*}}$ a heuristic genetic algorithm is considered and the solution process is:

1) Randomly generate *q* initial feasible solutions ΔR(i) such that the set of feasible solutions is denoted as a population and the initial population $\begin{array}{c} P_{0}=\left[\Delta R\left(1\right)\Delta R\left(2\right)...\Delta R\left(q\right)\right] \end{array}$,population size is 50.
2) The reliability threshold for the initial population is calculated and the results obtained are sorted from largest to smallest.
3) Select individuals with the highest fitness values to ensure that they will remain in the next generation. The individuals with the lowest fitness values are eliminated. Finally, a roulette algorithm is used to select the remaining individuals to determine which ones will be retained for the next step.
  4) The genetic diversity of the progeny can be controlled by setting the crossover probability *p*_*l*_ and the mutation probability *p*_*j*_. Let the crossover probability be 0.9 and the mutation probability be 0.1, the screened individuals were subjected to random cross-pairing and mutation operations. These newly generated individuals are then used to update the population and perform a new round of genetic manipulation.
5) Let the maximum number of iterations be 80. When the number of iterations reaches the initial set value, terminate the iterative operation and find the optimal solution set $\vec{\Delta R_{A}^{*}}$.

**Fig 8:**
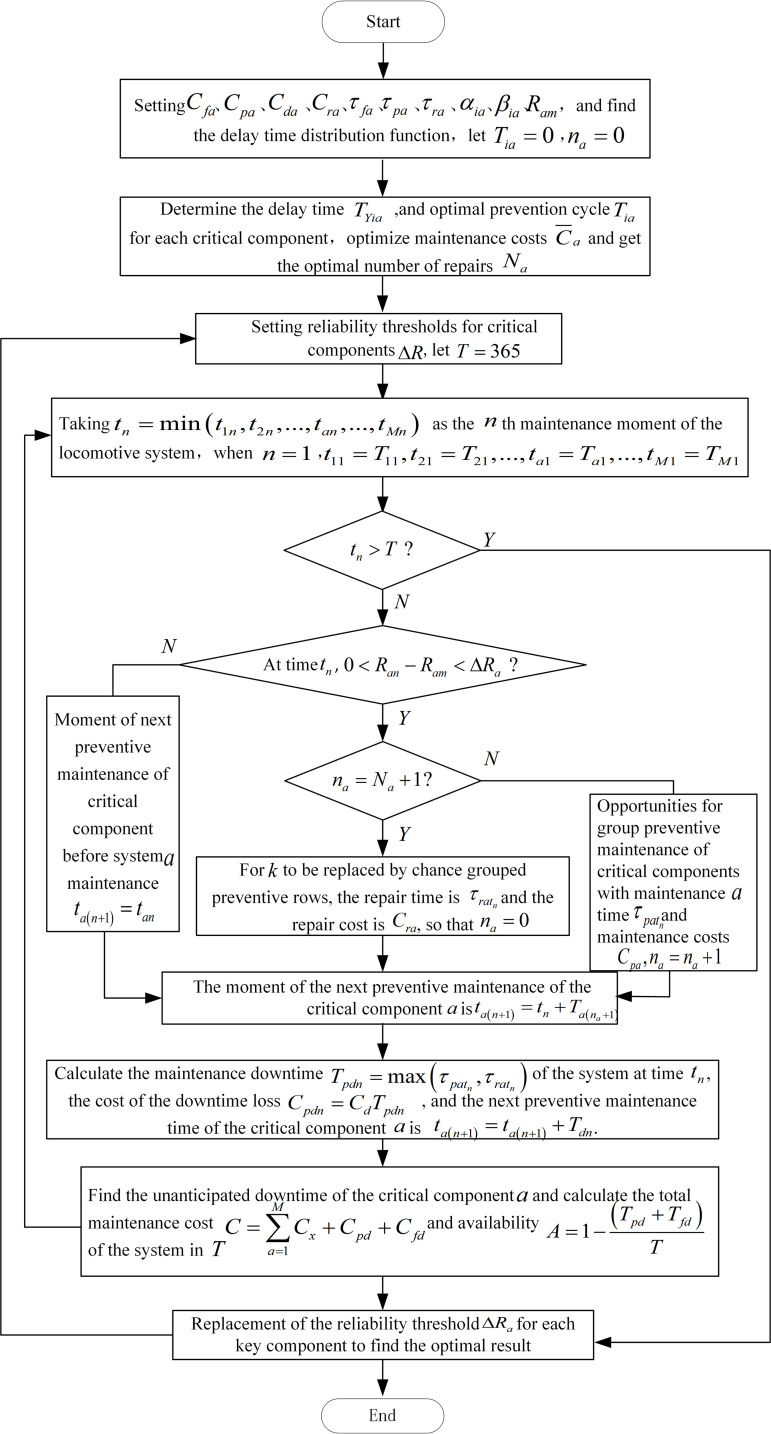
Flowchart of the optimization model solving algorithm.

The flowchart of the algorithm for solving the optimisation model of preventive maintenance plan for multi-component opportunity grouping is shown in Fig 8.

## Example analysis

We select five key components from the locomotive system that have a high maintenance frequency and are independent of each other (from S1 Fig and S1 Table), and that the locomotive will not operate properly if any one of the components fails. And the delay time function of each component obeys a Weibull distribution, i.e., $\lambda \left(t\right)=\left(\delta_{a}/\eta_{a}\right){\left(t/\eta_{a}\right)}^{\delta_{a}-1}\left(\delta_{a}>0,\eta_{a}>0\right)$. The effective operating cycle of the locomotive system is one year, i.e., T=365.The age reduction factor and the delay-time factor act simultaneously on each critical component, i.e., $\alpha_{a}=a=0.1$, $\beta_{a}=\beta =1.1$.The optimal preventive testing period for each critical component is set at 5, 7, 10, 12 and 15 days, respectively. Estimation of the relevant parameters of the components by means of field studies and historical empirical data. Let the average downtime loss $C_{da}$ per unit of time (one day) for a component of a locomotive be ¥10,000, and the other key components are shown in Table 2.

**Table 2:** Key Component related parameters.

Number	$\begin{array}{c} \beta_{a} \end{array}$	$\begin{array}{c} \eta_{a} \end{array}$	$\begin{array}{c} C_{fa} \end{array}$ /¥	$\begin{array}{c} C_{pa} \end{array}$ /¥	$\begin{array}{c} C_{ra} \end{array}$ /¥	$\begin{array}{c} C_{sa} \end{array}$ /¥	$\begin{array}{c} \tau_{fa} \end{array}$ /¥	$\begin{array}{c} \tau_{pa} \end{array}$ /¥	$\begin{array}{c} \tau_{ra} \end{array}$ /¥	$\begin{array}{c} R_{am} \end{array}$
1	2.0	30	120	50	1500	30	0.2	0.6	0.4	0.60
2	2.0	50	2500	350	5500	150	0.5	0.8	0.5	0.80
3	2.5	95	3600	650	8600	100	0.4	0.7	0.4	0.70
4	3.5	100	700	200	2800	90	0.5	0.6	0.5	0.6
5	3.0	140	450	150	1900	70	0.3	0.9	0.5	0.6

1) Solving for the optimal prevention cycle of a single component

By taking the data from the above table into Equation (18), we can obtain the relationship between the maintenance cost of each key component and the increase in the number of repairs, as shown in Table 3.

**Table 3:** Table of maintenance costs per unit of time for each component.

Component	Repair cost of components per unit of time $\begin{array}{c} {\overset{―}{C}}_{a} \end{array}$ ¥/day					Optimal number of preventive maintenance $\begin{array}{c} N_{a} \end{array}$
	1	2	3	4	5	
1	418.5	414.7	434.2	455.4	480.2	2
2	518.6	462.8	455.1	472.6	490.3	3
3	341.7	269.5	258.4	263.1	267.6	3
4	135.2	124.3	126.7	131.2	135.8	2
5	110.3	108.2	114.7	120.1	127.6	2

From Table 3, it can be seen that the maintenance cost per unit of time for each key component decreases and then increases as the number of repairs increases. Therefore, there exists a certain number of repairs at which the repair cost is minimized, and this number of repairs is the optimal number *Na* of repairs. Bringing each parameter into Equations (15) and (16), the delay time *T* of each key component can be obtained by association, as shown in Table 4.

**Table 4:** Delayed schedule for key components.

Component number	Individual critical component delay time$\begin{array}{c} T_{Yia} \end{array}$ /day			
	$T_{Y1a}$	$T_{Y2a}$	$T_{Y3a}$	$T_{Y4a}$
1	20.3	17.5	15.1	***
2	28.7	25.0	22.4	18.5
3	48.3	41.2	35.9	30.3
4	74.2	65.1	57.2	***
5	105.9	91.3	88.6	***

Combining the optimal inspection cycle for each critical component with Table 4 yields the optimal preventive maintenance cycle $T_{ia}$ for each critical component as shown in Table 5.

**Table 5:** Table of optimal preventive maintenance intervals for key components.

Component number	Optimal preventive maintenance cycles for individual critical components $\begin{array}{c} T_{ia} \end{array}$ /day			
	$T_{1a}$	$T_{2a}$	$T_{3a}$	$T_{4a}$
1	25.3	22.5	20.1	***
2	35.7	32.0	29.4	25.5
3	58.3	51.2	45.9	40.3
4	86.2	77.1	69.2	***
5	120.9	106.3	103.6	***

2) Multi-component scheduling Maintenance plan Solving

**Fig 9:**
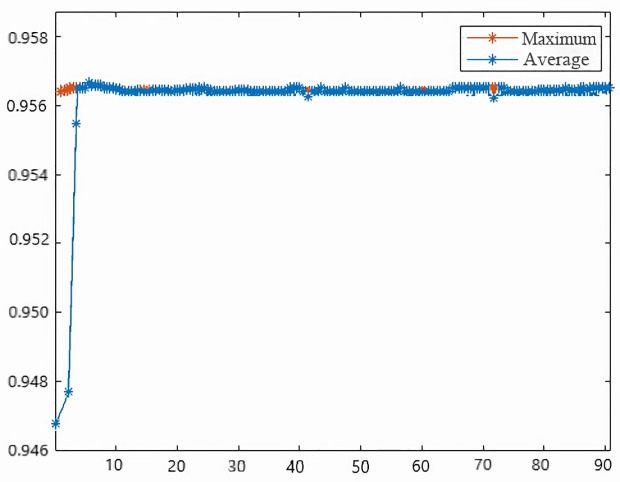
Iteration of maximum system availability.

Based on Equation (27) in the optimization model derived in the previous section, the reliability threshold for component repair is solved. The main parameters of the preset algorithm are shown in Table 6, and the iterative operations are performed in MATLAB software to obtain the results shown in Fig 9.

**Table 6:** Table of parameters of the genetic algorithm.

Main parameters	Initial population size	Maximum number of generations	probability of intersection	probability of mutation	Variable precision
Preset values	50	80	0.9	0.1	0.0001

According to the model solution can be obtained, the opportunity to group maintenance plan locomotive system availability is 95.63%, the maintenance cost is ¥256,347.2, and the reliability threshold of each key component is:


$$\vec{\Delta R_{A}^{*}}=\left(0.3625,0.2714,0.1820,0.1352,0.3186\right)$$ (32)

If the opportunity group maintenance program is not considered, the cost and availability of each key component to operate within the effective duty cycle T is determined from the data in Tables 3–5, as shown in Table 7. The results of considering and disregarding the use of opportunities for group maintenance were compared and the results are shown in Table 8.

**Table 7:** Maintenance costs and availability of key components and systems.

Component number	Running costs over cycle $\begin{array}{c} T \end{array}$	
1	118564.3	96.2%
2	121370.6	96.6%
3	65889.2	97.9%
4	33325.1	98.3%
5	30859.4	98.4%
systems	370008.6	88.0%

**Table 8:** Comparison of results of different repair strategies.

	Considering opportunities to group	Not considering opportunities to group
Number of preventive maintenance	13	40
Total maintenance costC /¥	256347.2	387179.17
usabilityA	95.63%	88.0%

Compared to the repair method that does not take into account chance grouping, all data are optimized to a greater extent after consideration. The specific performance is as follows: the number of preventive maintenance was significantly reduced during the locomotive operation cycle *T*, the total maintenance cost saved ¥130,831.97, the overhead saving rate reached 51.04%, and the system availability increased by 7.6%. It can be seen that this method has achieved better economic and social benefits. Through sensitivity analyses of key parameters, raising the reliability threshold from 0.85 to 0.95 would result in a 12.3% increase in total cost and a 4.7% increase in availability, indicating that thresholds need to be weighed against safety and economics. And the opportunity group maintenance strategy proposed in this paper shows unique advantages in terms of cost control and system availability. For example, compared with the traditional extended warranty model proposed by Cheng Zhonghua et al [5], significant savings in maintenance costs are achieved by dynamically triggering collaborative multi-component maintenance, which reduces the frequency of redundant maintenance. And compared to the Markov decision-based optimisation model proposed by Rui et al [12], the method dynamically adjusts the maintenance schedule through the reliability threshold, which significantly improves the overall locomotive availability within the same operating cycle. These improvements confirm the synergistic effect of the chance grouping strategy and the heuristic algorithm, and to some extent the superiority of the model.

A specific maintenance schedule based on the reliability thresholds is shown in Table 9.

**Table 9:** Maintenance schedule for each component cycle.

$\begin{array}{c} t \end{array}$ /day	Maintenance Methods					$\begin{array}{c} t \end{array}$ /day	Maintenance Methods				
	1	2	3	4	5	1	2	3	4	5	
25	M	M	N	N	N	201	R	M	M	N	N
47	M	M	M	N	N	224	M	M	N	R	M
67	R	M	N	N	N	248	M	M	M	N	N
92	M	R	M	M	N	268	R	R	N	N	N
116	M	M	N	N	M	293	M	M	M	N	N
136	R	M	M	N	N	315	M	M	N	M	N

Where M indicates that preventive maintenance is performed, N indicates that no preventive maintenance is performed, and R indicates that preventive replacement is performed. For example, at day 67, part 1 corresponds to R, meaning that preventive replacement is required; part 2 corresponds to M, meaning that preventive maintenance is required, and parts 3, 4, and 5 correspond to N, meaning that no maintenance activities are required. A check of the entire schedule shows that there is at least one component that does not need to be overhauled in every scheduling maintenance activity, so scheduling maintenance is effective in saving downtime and maintenance overhead compared to traditional overhaul processes.

## Conclusion

This paper focuses on the problem of optimizing the C4 repair operation process of harmonious electric locomotives, and proposes a set of systematic solutions from problem analysis to model construction, and then to algorithm design and case study.

1) The inspection cycle optimization model for single components is developed with availability and reliability as constraints under the consideration of minimum maintenance cost. It also considers the effect of maintenance improvement factors on component failure rates to optimize single-component preventive maintenance cycles.
2) Opportunistic grouping is performed according to the reliability threshold, and the preventive maintenance schedule optimization model for multiple components is established with the objective of maximum availability, solved using genetic algorithm and verified with examples. According to the calculation results, it can be seen that the total cost of the same locomotive system can be saved by 51.04% and the availability can be increased by 7.89% in the same operation cycle under the preventive maintenance method considering the use of opportunity grouping.
3) The development of a specific maintenance schedule based on the reliability thresholds demonstrates the effectiveness of opportunity grouping maintenance in saving downtime and maintenance overhead compared to the traditional overhaul process.

The effectiveness of this model has been verified in the case of the Y locomotive depot, but further evaluation is required for large-scale extension to different locomotive maintenance environments, such as differences in maintenance record formats and data accuracy between depots, which may affect the accuracy of parameter calibration. A uniform locomotive O&M database needs to be established and data cleaning specifications developed to ensure consistency of model inputs. In areas of high cold or humidity, the rate of component degradation can be significantly accelerated, requiring the introduction of environmental correction factors to dynamically adjust maintenance intervals.

Therefore, the multi-part opportunity group preventive maintenance model optimization has a certain feasibility, which provides methodological support for simplifying the locomotive maintenance operation steps, reducing the maintenance downtime costs, and improving the maintenance efficiency. Inter-component coupling failures are not considered in this study and the parameters are dependent on historical data quality. In the future, the dynamics of the model can be improved by combining real-time sensor data, investigating the effect of functional coupling between components on the maintenance strategy, and developing a dynamic optimisation model based on real-time monitoring data.

## Supporting information

S1 File Operational time for each step in the main C4 repair process. (XLSX)

S1 Fig Maintenance data for a type of electric locomotive in 2023. (TIF)

S1 Table Repair of some key components of an electric locomotive type in 2023. (DOCX)
