# Supplementary figures and images for: Research on optimization of C4 repair operation of Harmony electric locomotive based on preventive maintenance
Source: PLoS One. 2025 Jul 24;20(7):e0328399. doi: 10.1371/journal.pone.0328399 (PMC12289093; doi:10.1371/journal.pone.0328399)

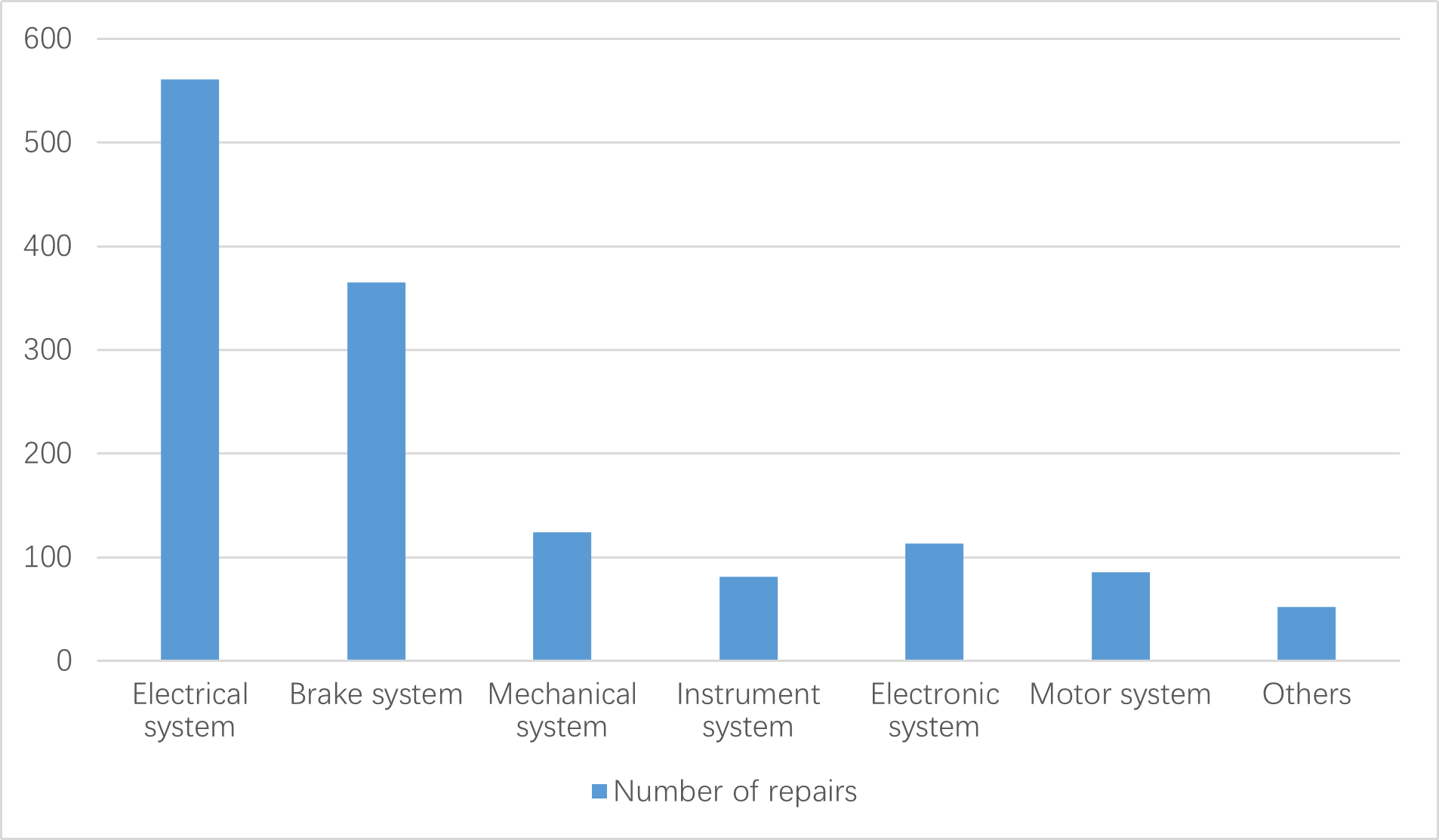

Supplement: S1 Fig — (TIF) [file pone.0328399.s002.tif]
