# Supplementary material for: Research on optimization of C4 repair operation of Harmony electric locomotive based on preventive maintenance
Source: PLoS One. 2025 Jul 24;20(7):e0328399. doi: 10.1371/journal.pone.0328399 (PMC12289093; doi:10.1371/journal.pone.0328399)
Supplement: S1 Table — (DOCX) [file pone.0328399.s003.docx]

##### S1 Table. Maintenance of some major components of a locomotive type in 2023

| Subsystems | Fault content | Number of items |
| --- | --- | --- |
| Electrical systems | Damaged pantograph spring element | 26 |
|  | Poor contact between slide plate and bow angle | 15 |
|  | Pantograph slider breaks | 7 |
|  | Main circuit breaker failure | 31 |
|  | Electromagnetic valve air leakage | 14 |
| Brake system | Compressor fan jammed | 34 |
|  | Compressor oil leakage | 12 |
|  | Air brake valve failure | 46 |
| Mechanical systems | Oil leakage of oil pressure shock absorber | 36 |
|  | Wheel nozzle blockage | 27 |
| Motor system | Split camera failure | 42 |
|  | Display failure | 12 |
| Instrument | Current meter failure | 17 |
| Else | ... | 49 |
